# The Impact COVID-19 Infection on Cancer Patients: A Tertiary Cancer Center Experience in Jordan

**DOI:** 10.7759/cureus.51310

**Published:** 2023-12-29

**Authors:** Kamal Al-rabi, Fadwa Al-Qadi, Akram Al-Ibraheem, Khalid Halahleh, Samer Salah, Hazim Ababneh, Mohammad Akkawi, Maher Sughayer, Lana Tafesh, Layan Abu Abed, Mohammad Ma'koseh

**Affiliations:** 1 Medical Oncology, King Hussein Cancer Center, Amman, JOR; 2 Internal Medicine, School of Medicine, University of Jordan, Amman, JOR; 3 Internal Medicine, King Hussein Cancer Center, Amman, JOR; 4 Nuclear Medicine, King Hussein Cancer Center, Amman, JOR; 5 Pathology and Laboratory Medicine, King Hussein Cancer Center, Amman, JOR

**Keywords:** jordan, mortality, systemic anitcancer treatment, cancer, covid-19

## Abstract

Background: Cancer patients are at higher risk of serious complications of COVID-19. Few studies evaluated the impact of COVID-19 on cancer patients in low- and middle-income countries. Our study aims to evaluate the outcomes of COVID-19 infection in cancer patients treated at our institution.

Methods: Medical records of patients with a positive COVID-19 polymerase chain reaction (PCR) between April 2020 and October 2020 were reviewed. Fisher's exact test and logistic regression analysis were employed to correlate various variables with mortality. Survival estimates were generated using the Kaplan-Meier method.

Results: A total of 317 patients were included, with a median age was 55 years (range: 19-88). 82 (25.9%) had hematological neoplasms while the remainder had solid cancers. At the time of infection, 220 (69.4%) had active cancer, and 99 (31.2%) had received systemic anticancer treatment (SACT) within four weeks. Hospitalization was required for 101 (31.8%), 17 (5.3%) were admitted to the ICU and 50 (15.8%) died. Among patients with active cancer, SACT was delayed or discontinued in 140 (63.6%) patients.

In the entire patient cohort, low albumin (p=<0.001) and leucocytosis (p=<0.001) correlated with mortality within six months of COVID-19 infection. The six-month mortality rate in patients with active cancer was significantly higher in patients with hypertension (p=0.024), no recent SACT (0.017), hematological cancer (p=0.029), low albumin (p=<0.001), leucocytosis (p=0.002) and lymphocyte count of less than 500/µL (p=0.004). Recent chemotherapy was associated with better 6-month survival rates (78.8% vs 89.9%, p=0.012) in patients with active cancer, patients with solid cancers (95.9% vs 82.2%, p=0.006) and was non-inferior in patient with hematological neoplasms (72% vs 65.4%, p=0.519).

Conclusion: COVID-19 infection in our cancer patients was associated with significant morbidity and mortality and adversely affected their treatment. The decision to delay or discontinue SACT should be individualized, considering other risk factors for mortality.

## Introduction

SARS-CoV-2, first identified in December 2019, triggered a global pandemic of a respiratory illness known as COVID-19 [1]. The pandemic resulted in significant morbidity and mortality including 6.9 million deaths [2]. In addition, many aspects of healthcare delivery were disrupted, and hospitals and ICUs were overwhelmed. 

Cancer patients are more susceptible to getting infected with SARA-CoV-2, and they are at higher risk for COVID-19 infection-related morbidity and mortality as they are generally older, have coexisting comorbidities and immunocompromised because of their underlying malignancy or due to systemic anticancer treatment (SACT) [3-5]. 

Forecasting the progression of COVID-19 in cancer patients presents a challenge due to the variable extent of additional health decline. A meta-analysis encompassing data from 20 distinct studies revealed a broad range in COVID-19 related mortality among cancer patients, varying from 5.9% to 50% [6]. Beyond age and comorbidities, various cancer-related variables were identified as associated with increased mortality. For instance, Lee et al. reported that patients with hematological malignancies infected with COVID-19 exhibited a worsened clinical condition and an increased risk of death compared to those without COVID-19 infection [7]. In another study, both hematological malignancies and lung cancer patients had increased risk of severe COVID-19 infection [8]. Additionally, Kong et al. reported that patients with lung adenocarcinoma were more susceptible to SARS-CoV-2 infection than patients with lung squamous cell carcinoma [9]. 

Moreover, the effect of recent SACT on the outcomes of COVID-19 infection in cancer patients remains unclear. While initial studies suggested an association between SACT administration and an increased risk of death due to COVID-19, recent studies have found no adverse effects of recent SACT [8,10-14].

Few studies reported the outcomes of cancer patients infected with SARS-CoV-2 in low- and middle-income countries. Jordan is a middle-income country with limited resources [15]. The King Hussein Cancer Center is a tertiary healthcare center that provides care for about 60% of cancer cases in Jordan [16]. Herein, we report the outcomes of cancer patients infected with SARS-CoV-19 at our center. 

## Materials and methods

Files and medical records of adult cancer patients (>18 years) with a positive polymerase chain reaction (PCR)-confirmed SARS-CoV-2 infection from April 2020 to October 2020 were retrospectively reviewed. The data included both in-patients and out-patients. Various demographic, cancer, and therapy-related variables, along with initial laboratory tests at the time of COVID-19 diagnosis, the need for hospitalization, and ICU admission were collected. Indications for hospitalization included hypoxia and/or hemodynamic instability. Patients with metastatic neoplasm and those receiving SACT were considered to have active cancer. Recent SACT was defined as administration of systemic chemotherapy, hormonal therapy, target therapy, or immunotherapy within four weeks of COVID-19 diagnosis. Mortality was defined as death within six months of COVID diagnosis. Delayed SACT was defined as postponing SACT for more than one week. 

Patient characteristics were tabulated and presented in numbers and percentages. The correlation between different variables and mortality was done using Fisher’s exact test. The variables found to have significant association with mortality (p<0.05) by univariate analysis, were subsequently entered into the multivariate logistic regression model. Survival was calculated from the time from the diagnosis of COVID 19 infection till the last follow-up, and was estimated using the Kaplan-Meier method.

The study was approved by the King Hussein Cancer Centre Institutional Review Board (IRB) in compliance with the principles of the Declaration of Helsinki. A waiver of the written informed consent was granted due to the retrospective nature of the review, and the data were anonymized and maintained with confidentiality. 

## Results

Patients’ characteristics

A total of 317 patients were included with a median age of 55 (range: 19-88) years and an almost equal gender distribution. The PCR test was indicated in 191 individuals (60.3%) presenting COVID-19-related symptoms. For the remaining patients, testing was conducted electively, either before scheduled surgeries or elective procedures, or after potential exposure to COVID-19-infected individuals. A total of 235 patients (74%) had solid malignancies, whereas 82 (25.9%) had hematological neoplasms. The most common cancer diagnosis was breast cancer (n=88, 27.8%) and the most common hematological neoplasm was lymphoma (n=31, 42.5%). On the time of positive PCR for SARS-CoV-2, 220 (69.4%) patients had an active cancer; among those, 170 (77%) had solid malignancies and 50 (23%) had haematological neoplasms. Of the 97 (30.6%) patients who were in remission, 65 (67%) had solid tumors, whereas 32 (33%) had hematological neoplasm. Patients’ characteristics are detailed in Table 1. 

**Table 1:** Patient’s characteristics SACT: Systemic anticancer treatment, WBC: White blood cell, ALT: Alanine aminotransferase, AST: Aspartate aminotransferase. *Other comorbidities not included in the list.

Characteristic	Number (%)
Male	142 (44.8%)
Female	175 (55.2%)
Age > 60	116 (36.6%)
BMI > 30	139 (43.9%)
Current or ex-smoker	130(41%)
Comorbidities	197 (62.1%)
Diabetes mellitus	90 (28.4%)
Hypertension	121 (38.2%)
Cardiovascular disease	39 (12.3%)
Chronic obstructive lung disease	10 (3.2%)
Chronic kidney disease	21 (6.6%)
Other comorbidities*	27 (8.5%)
Solid cancer	235 (74.1%)
Hematological neoplasm	82 (25.9%)
Active cancer	220 (69.4%)
Lung cancer	16 (5%)
Breast cancer	88 (27.8%)
Gastrointestinal cancers	47 (14.8%)
Genitourinary cancer	35 (11%)
Head and neck cancer	16 (5%)
Gynaecological cancer	14 (4.4%)
Sarcoma	6 (1.9%)
Non-Hodgkin lymphoma	26 (8.2%)
Hodgkin lymphoma	9 (2.8%)
Leukaemia	26 (8.2%)
Multiple myeloma	19 (6%)
Other cancers	15 (4.7%)
No SACT within 4 weeks	218 (68.8%)
Haemoglobin < 10 grams/dl	86 (27.2%)
WBC count > 11/µl	107 (33.7%)
WBC count < 4/µl	110 (34.7%)
Neutrophil count < 1,000/µl	49 (15.4%)
Lymphocyte count < 1,000/µl	198 (62.4%)
Lymphocyte count < 500/µl	85 (26.8%)
High ALT	27 (8.5%)
High AST	38 (12%)
High bilirubin	21 (6.6%)
Albumin ≤ 3.5 grams/dl	130 (41%)

Survival outcomes

**Figure 1:**
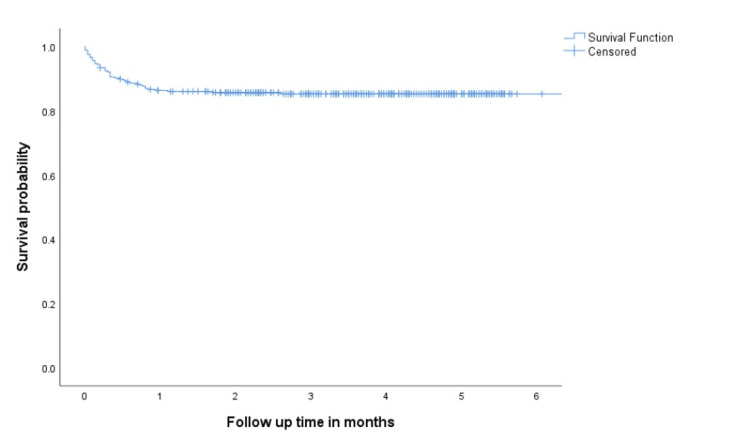
Overall survial in all patients

During the study period, 101(31.9%) patients were hospitalized, 17 (5.3%) were admitted to ICU and 50 (15.8%) have died. The six-month survival rate for the entire cohort was 84.2% (Figure 1). The mortality rate at one month was 14.7%. Notably, in patients who required hospitalization and ICU admission, 42 (41.6%) and 15 (88.2%) died during the hospitalization, respectively.

Compared to patients who stayed alive, the group of patients who died within six months of COVID-19 infection had a higher frequency of male gender (p=0.018), age > 60 years (p=0.001), diabetes (p=0.008), hypertension (p=0.005), chronic kidney disease (p≤0.001), hematological neoplasms (p=0.005), abnormal serum aspartate aminotransferase (AST) (p=0.038), low albumin (p≤0.001), hemoglobin < 11 g/dl (p=0.017), leucocytosis (p≤0.001) and lymphocyte count <500 (0.004) (Table 2). 

**Table 2:** Univariate analysis for overall mortality in all patients WBC: White blood cell; ALT: Alanine aminotransferase; AST: Aspartate aminotransferase; SACT: Systemic anticancer treatment

Variable	Alive %	Dead %	P value
Male	40%	58.1%	0.018
Female	60%	41.9%	
Age ≤ 60	67.4%	42%	0.001
Age > 60	32.6%	58%	
BMI ≤ 30	56.9%	53.1%	0.617
BMI > 30	43.1%	46.9%	
Current or ex-smoker	60%	51.2%	0.297
Never smoked	40%	48.2%	
Diabetes mellitus	25.5%	44%	0.008
No diabetes mellitus	74.5%	56.%	
Hypertension	34.5%	66%	0.005
No hypertension	65.5%	44%	
Cardiovascular disease	10.9%	20%	0.071
No cardiovascular disease	89.1%	80%	
Chronic lung disease	3%	4%	0.709
No chronic lung disease	97%	96%	
Chronic kidney disease	4.1%	29%	<0.001
No chronic kidney disease	95.9%	80%	
Other comorbidities	9.7%	2%	0.095
No other comorbidities	90.3%	98%	
Solid tumour	77.2%	58%	0.005
Hematological tumour	22.8%	42%	
Lung cancer	4.9%	6%	0.737
Other cancer	95.1%	94%	
Active cancer	68.2%	76%	0.27
Remission	31.2%	24%	
Haemoglobin ≥ 10 g/dl	78.1%	59.6%	0.017
Haemoglobin < 10 g/dl	21.9%	40.4%	
WBC count > 11/µl	5.3%	38.3%	<0.001
WBC count ≤ 11/µl	94.7%	61.7%	
WBC count ≥ 4/µl	59.6%	78.7%	0.021
WBC count < 4 /µl	40.4%	21.3%	
Neutrophil count ≥ 1,000/µl	82.5%	89.1%	0.293
Neutrophil count < 1,000/µl	17.5%	10.9%	
Lymphocyte count ≥ 1,000/µl	39.5%	32.6%	0.417
Lymphocyte count <1000/µl	60.5%	67.4%	
Lymphocyte count ≥ 500/µl	78.9%	56.5%	0.004
Lymphocyte count < 500/µl	21.1%	43.5%	
Normal ALT	94.1%	85.1%	0.073
High ALT	5.9%	14.9%	
Normal AST	91.1%	78.7%	0.036
High AST	8.9%	23.3%	
Normal bilirubin	96%	87.2%	0.075
High bilirubin	4%	12.8%	
Albumin ≤ 3.5 g/dl	75.2%	21.3%	<0.001
Albumin >3.5 g/dl	24.8%	78.7%	
Impact on SACT	41.5%	77.6%	<0.001
No impact on SACT	58.5%	22.4%	

On multivariate analysis, low albumin (p≤0.001) and leucocytosis (p≤0.001) correlated with mortality within six months of COVID-19 infection (Table 3).

**Table 3:** Multivariate analysis for mortality in all patients AST: Aspartate aminotransferase

Variable	Hazard ratio	95% confidence interval		P value
		Lower	Upper	
Male gender	.988	.337	2.899	0.982
Age > 60 years	1.374	.452	4.177	0.575
Diabetes mellitus	2.388	.769	7.412	0.132
Hypertension	2.505	.772	8.135	0.126
Chronic kidney disease	1.283	.269	6.132	0.754
Hematological neoplasm	2.336	.585	9.322	0.064
High AST	2.336	.585	9.322	0.23
Low albumin	14.926	4.745	46.954	< 0.001
Leucocytosis	11.992	3.167	45.416	< 0.001
Lymphocyte count < 500/µl	2.657	.883	7.990	0.082
Hemoglobin < 10 g/dl	.721	.236	2.204	0.566

In the group of patients with active cancer (n=220), the six-month survival rate was 83.1%. In this group, patients who died within six months of COVID-19 infection, compared to survivors, were more likely to be males (p=0.04); older than 60 years (p=0.01); and have hypertension hypertension (p=0.023), chronic kidney disease (p=0.019), hematological neoplasms (p≤0.001), low albumin (p≤0.001), hemoglobin < 10 g/dl (p=0.017), leucocytosis (p≤0.001), lymphocyte count < 500/µl (p=0.004); and have not received recent SACT (p=0.01) (Table 4).

**Table 4:** Univariate analysis for overall mortality in patients with active cancer SACT: Systemic anticancer treatment; WBC: White blood cell; ALT: Alanine aminotransferase; AST: Aspartate aminotransferase.

Variable	Alive %	Dead %	P value
Male	40.1%	57.9%	0.04
Female	59.9%	42.1%	
Age ≤ 60 years	60.2%	47.4%	0.01
Age > 60 years	30.8%	52.6%	
BMI ≤ 30 kg/m^2^	57.5%	57.9%	0.97
BMI > 30 kg/m^2^	42.5%	42.1%	
Current or ex-smoker	60.7%	54.8%	0.55
Never smoked	39.3%	45.2%	
Diabetes mellitus	22%	36.8%	0.053
No diabetes	78%	63.2%	
Hypertension	30.8%	50%	0.023
No hypertension	69.2%	50%	
Cardiovascular disease	11%	15.8%	0.404
No cardiovascular disease	89%	84.2%	
Chronic lung disease	2.2%	2.6%	0.87
No chronic lung disease	97.8%	97.4%	
Chronic kidney disease	4.4%	15.8%	0.019
No chronic kidney disease	95.6%	84.2%	
Other comorbidities	11%	2.6%	0.111
No other comorbidities	89%	97.4%	
Solid cancer	81.9%	55.3%	<0.001
Hematological neoplasm	18.1%	44.7%	
Lung cancer	5.5%	5.3%	0.954
Other cancers	94.5%	94.7%	
No SACT within 4 weeks	51.1%	73.3%	0.025
SACT within 4 weeks	48.9%	26.7%	
Haemoglobin ≥ 10 g/dl	77.2%	56.8%	0.02
Haemoglobin < 10 g/dl	22.8%	43.2%	
WBC count > 11/µl	97.8%	64.9%	<0.001
WBC count ≤ 11/µl	2.1%	35.1%	
WBC count ≥ 4/µl	43.5%	18.9%	0.009
WBC count < 4 /µl	56.5%	81.1%	
Neutrophil count ≥ 1,000/µl	80.4%	88.9%	0.254
Neutrophil count < 1,000/µl	19.6%	11.1%	
Lymphocyte count ≥ 1,000/µl	40.2%	27.8%	0.189
Lymphocyte count < 1000/µl,	59.8%	67.2%	
Lymphocyte count ≥ 500/µl	78.3%	52.8%	0.004
Lymphocyte count < 500/µl	21.7%	47.2%	
Normal ALT	92.9%	86.5%	0.261
High ALT	7.1%	13.5%	
Normal AST	90.5%	78.4%	0.07
High AST	9.5%	21.6%	
Normal bilirubin	95.2%	86.5%	0.091
High bilirubin	4.8%	13.5%	
Albumin > 3.5 g/dl	77%	16.2%	<0.001
Albumin ≤ 3.5 g/dl	23%	73.8%	

On multivariate analysis, hypertension (p=0.024), no recent SACT (p=0.017), hematological malignancy (p=0.029), low albumin (p≤0.001), leucocytosis (p=0.002) and lymphocyte count < 500/µl (p=0.004), correlated with mortality within six months of COVID-19 infection (Table 5).

**Table 5:** Multivariate analysis for mortality in patients with active cancer SACT: Systemic anticancer treatment

Variable	Hazard ratio	95% confidence interval		P value
		Lower	Upper	
Male gender	1.255	.311	5.062	0.749
Age > 60 years	1.166	.276	4.919	0.834
Hypertension	6.252	1.271	30.744	0.024
Chronic kidney disease	1.169	.113	12.113	0.896
Hematological neoplasm	6.198	1.200	32.021	0.029
Low albumin	35.881	6.977	184.533	< 0.001
Leucocytosis	19.745	2.996	130.114	0.002
Lymphocyte count < 500/µl	13.386	2.298	77.978	0.004
Hemoglobin < 10 g/dl	1.225	.303	4.951	0.776
Recent SACT	7.345	1.431	37.698	0.017

Effect of recent administration of SACT

**Figure 2:**
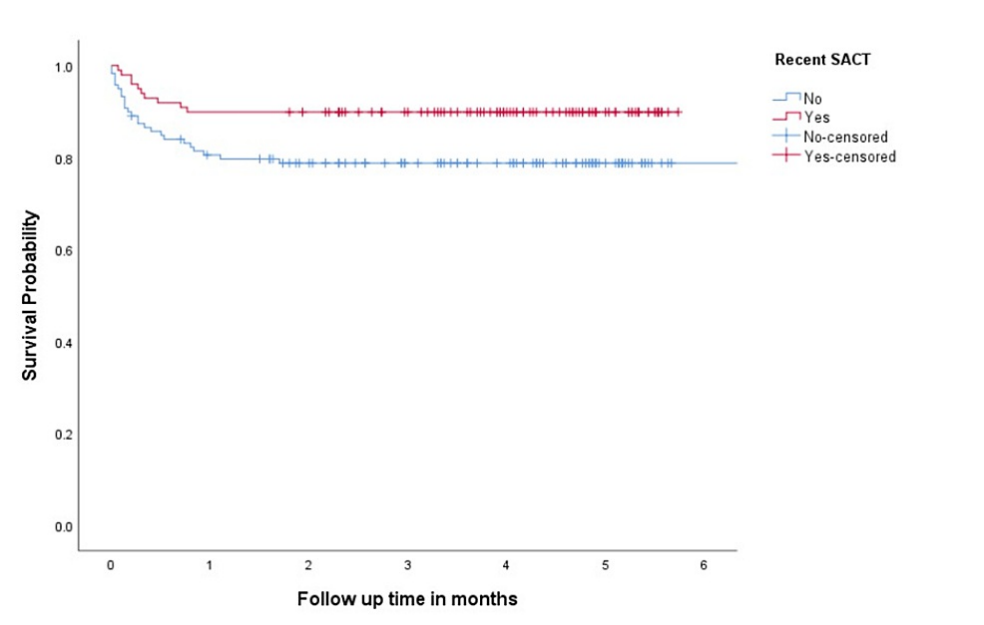
Overall survival in patients with active cancer according to recent SACT SACT: Systemic anticancer treatment

In patients with active cancer (n=220), 99 (45%) received SACT within four weeks of COVID-19 infection. Types of SACT were chemotherapy in 64 (29%), chemoimmunotherapy in 16 (7.2%), immunotherapy in 12 (5.4%), target therapy and hormonal therapy in 5 (2.2%) and 2 (0.9%) patients, respectively. Compared to patients with no recent SACT, patients who received recent SACT had better six-month survival rates (78.8% vs 89.9%, p=0.012), figure (2).

In patients with hematological neoplasm (n=50), the six-month survival rates were comparable between the two groups (64.5% vs 72%, p=0.519), bur in patients with solid tumours, the six-month survival rate was superior in patients who received recent SACT compared to patients who did not (95.9% vs. 82.2%; p=0.006).

**Figure 3:**
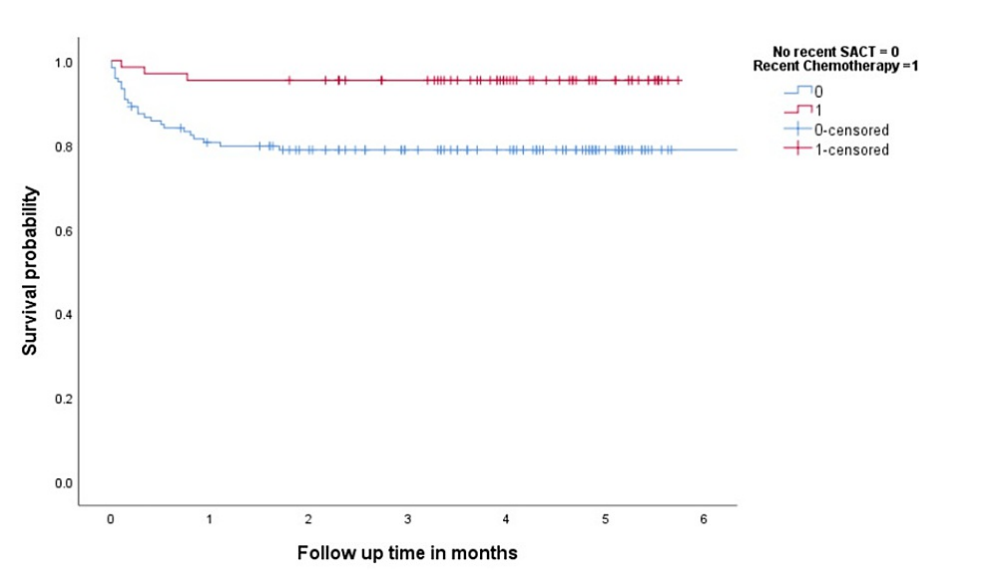
Overall survival in patients with active cancer according to recent systemic chemotherapy compared to patients with no recent SACT SACT: Systemic anticancer treatment

We also evaluated the effect of different types of SACT on survival. The 6-month survival rate was inferior in patients who did not receive recent SACT compared to patients who received recent chemotherapy (78.8% vs. 95% %, p=0.04) (Figure 3). However, there was no significant difference in the six-month survival rates between patients who received chemoimmunotherapy and those who did not receive recent SACT (75% vs. 78.8%, respectively; p=0.747). 

Impact of COVID-19 infection on the administration of SACT

Among patients with active cancer (n= 220), timely administration of SACT was affected in 140 (63.6%) patients because of COVID-19 infection. SACT was delayed in 105 (47.7%) patients and permanently discontinued in 35 (15.9%) patients. 

In 50 patients who had an active hematological neoplasm, SACT administration was delayed in 28 (56%) and permanently discontinued in 13 (26%). The delay was because of hospitalization in 15 (53.6%) patients and due to positive PCR in the remaining patients. 

On the other hand, in patients with active solid cancers (n=170), SACT administration was delayed in 77 (45.3%) and permanently discontinued in 22 (12.9%). The delay was because of hospitalization in 17 (22.1%) patients and due to positive PCR in the remaining patients. 

## Discussion

The COVID-19 pandemic has had profound effects on healthcare services worldwide, and Jordan is no exception. Cancer, a complex group of diseases, relies on timely diagnosis, intervention, and treatment for better prognosis. However, cancer patients, already with weakened immunity, face heightened risks of infectious morbidities. In this study, we explore the impact of the pandemic on cancer patients within the context of limited resources and the unavailability of COVID-19-directed antiviral and antibody treatments.

The overall mortality rate in our study (15.8%) is comparatively lower than the 28% reported by the United Kingdom Coronavirus Cancer Monitoring Project (UKCCMP) study [7]. Notably, our study included all patients (out-patients and hospitalized patients) with a positive PCR test, diverging from the UKCCMP study that focused solely on hospitalized patients. However, the mortality rate among hospitalized patients (41.6%) was higher in our study compared to the UKCCMP study and the Lean European Open Survey on SARS-CoV-2 Infected Patients (LEOSS) registry data [16]. This discrepancy may stem from differing admission criteria and the relative lack of supportive healthcare resources in our setting.

An important observation is the variation in factors independently correlating with mortality between patients with active cancer and the entire patient cohort. While leucocytosis and hypoalbuminemia were independent correlates in the overall patient group, patients with active cancer exhibited independent correlations with other variables, including the diagnosis of hematological neoplasms, lymphopenia and hypertension. It has been reported that COVID-19 immunological signature and post-viral clearance immune state are similar between patients with solid tumors and non-cancer patients [17]. On the other hand, patients with hematological cancer have an exhausted immune response to COVID-19 infection due to lower CD8+ve T cells and B cell aplasia [12]. This may explain the higher impact of impaired immunity on the outcomes in this group.

A significant challenge during the pandemic is the timely administration of SACT. This decision must consider infection severity, cancer type, and treatment goals. Interestingly, we observed a higher six-month mortality rate among patients who did not receive recent SACT. Our findings align with data from the Memorial Sloan Kettering Cancer Centre and the recent UKCCMP study, which also demonstrated that recent chemotherapy was associated with improved six-month survival rates in patients with active cancer and solid tumors [11,12]. Changes in the practice during the pandemic may have resulted in the selection of fitter patients and those being treated with the curative intention to continue SACT. On the other hand, recent SACT was reported to be associated with higher mortality rates in COVID-19-infected cancer patients [9,10]. Notably, this reduction in mortality rates with SACT was not observed in patients with hematologic malignancies, highlighting the need for personalized decision-making based on multiple factors.

The pandemic's impact on the administration of SACT, as reported in our study, is consistent with previous findings [17-20]. Delays in chemotherapy administration, as high as 65% in one study, and delays in chemotherapy, radiotherapy, or surgery in 29-44% of patients in another study, emphasize the challenges faced by cancer patients [19,21]. Patient involvement in critical decisions, as seen in a survey among cancer patients in Lebanon, underscores the importance of considering patients' preferences [22].

Since its discovery in December 2019, several treatments have been approved for managing COVID-19 infections, demonstrating efficacy in cancer patients. In May 2020, remdesivir became the inaugural antiviral agent sanctioned for treating severe COVID-19 infections [23]. A study involving 222 patients with active cancer revealed an 80% reduction in mortality associated with the early use of remdesivir [24]. Neutralizing antibodies, bamlanivimab and etesevimab, targeting the SARS-CoV-2 surface spike glycoprotein, were isolated from convalescent plasma, resulting in decreased hospitalization and mortality rates among high-risk ambulatory patients [25]. Bamlanivimab was particularly effective in reducing hospital and ICU admissions and preventing hypoxia in cancer patients with mild-to-moderate COVID-19 infections [26]. Nirmatrelvir plus ritonavir (Paxlovid) demonstrated an 89% reduction in progression to severe COVID-19 infections in high-risk patients during the Evaluation of Protease Inhibition for Covid-19 in High-Risk Patients (EPIC-HR) trial [27]. A report from the EPICCOVIDEHA registry indicated that COVID-19 patients with hematological malignancies treated with Paxlovid had a lower 30-day mortality (2% vs. 11%) compared to other COVID-19-directed treatments [28]. In the phase 3 component of the MOVe-OUT placebo-controlled trial, oral molnupiravir reduced the risk of hospitalization and death in high-risk patients with mild-to-moderate COVID-19 infections [29]. A matched retrospective study found the efficacy of molnupiravir comparable to Paxlovid in immunocompromised cancer patients [30].

While vaccination remains the paramount and effective preventive measure against SARS-CoV-2 infection, observational studies suggest a potentially blunted serological response to vaccines in cancer patients. This is primarily attributed to the immunosuppressive state of the cancer type and acquired immunodeficiency from anticancer treatments [31,32]. Nevertheless, vaccination is strongly recommended in this group, offering partial protection by inducing long-term T-cell-mediated immunity, irrespective of antibody titers, thereby reducing the risk of breakthrough infections, severe diseases, and hospitalization [33]. Among cancer patients, the mRNA 1237 vaccine appears to be the most immunogenic, followed by BNT162b2 and Ad26.COV2.S [34]. For those with lower antibody titers after the initial vaccine dose, an additional dose may be necessary to enhance immunogenicity [33,34].

Despite being one of the few reports addressing the outcomes and impacts of the COVID-19 pandemic on cancer patients in low- and middle-income countries, we acknowledge the limitations of our retrospective design and the relatively short follow-up time.

## Conclusions

COVID-19 infections in our cancer patients resulted in significant morbidity and mortality, negatively impacting the administration of SACT. Recent chemotherapy administration was associated with lower mortality in patients with solid tumors but did not affect the survival of patients with hematological neoplasms. Decisions to delay the administration of SACT in the context of future pandemics should approached with careful consideration.
